# Supplementary material for: “The Ability to Go Out into the World Is the Most Important Thing”—A Qualitative Study of Important Exercise Outcomes for People with Lung Cancer
Source: Curr Oncol. 2024 Jan 29;31(2):733–46. doi: 10.3390/curroncol31020054 (PMC10887680; doi:10.3390/curroncol31020054)
Supplement: Supplementary file 1 [file curroncol-31-00054-s001.zip › curroncol-2808364-supplementary.pdf]

Supplementary Materials

Section S1: Question Guide

Introduction

Introduction, welcome to country, introduction of facilitators, and housekeeping.

Consent

Verbal consent sought from participant(s) to commence recording. Reminder that once we start recording we will ask each if they give consent to being included in the study. (**Start recording**). Verbal consent to participate in study confirmed.

Further introduction and definition on outcome measures

The overarching aim of this interview is to hear about your experiences, opinions and priorities regarding outcome measures (and I will go over than in a second) and that could be prior to, during and following exercise pre- and rehabilitation programs.

An outcome measure is a broad term – referring to any assessment that can be used to measure your current level of health, function or movement. Examples of outcome measures are: measuring blood pressure, assessing physical tasks like walking or answering questions about symptoms, experiences or feelings.

An outcome measure may be collected prior to starting, during or after exercise. An outcome measure can help exercise professionals decide if and what exercise is safe for a person to complete, and, can help exercise professionals understand what each person needs during the program and to see if things change during the program.

Some of you may have completed an exercise program (or the person you care for may have) after being diagnosed with lung cancer- if that is the case we encourage you to draw on your experiences. It may also be the case that you or the person you care for has not yet been involved in an exercise program. If this is your situation we would like you to think about the reasons that might make you decide to start exercising during your lung cancer journey.

Section S1.1 – Physical activity

Seeding question:

1a. Following the initial diagnosis with lung cancer, were there activities or physical tasks that you found difficult as someone diagnosed with lung cancer, or as a carer of someone with lung cancer what was your perception of what was difficult?

Additional background and focused question:

Background: Exercise staff often measure things like strength and/or fitness. An example of a strength test may be lifting a heavy weight, or testing you standing up and sitting down from a chair. An example of a fitness test could be a walking test or an activity that makes you feel breathless. (end slide)

1b. 'Which physical measures are important to you?

Prompts: Why is that?

1c. Are there other physical aspects you have difficulty with that you think exercise staff should measure before, during, or after exercise?'

Section S1.2 Body structure and function

Seeding questions:

2a. What symptoms did you experience as a result of your lung cancer or treatment?

Additional background and focused question:

Background: As we mentioned earlier - Exercise professionals may ask a single question or series of questions to gather information. Questions can ask about symptoms, feelings or experiences. This may be in the form of a single question where you rate a single symptom or a questionnaire where you are asked to rate multiple things over a set period of time on a scale as you see in the pictures.

2b. Thinking about what is important to you, which of your symptoms do you think that exercise professionals should measure before, during or after exercise?

2c. Are there any other symptoms or feelings which could affect your ability to exercise that we have not discussed?

Section S1.3 Participation

Focused Questions:

3a. Lung cancer can impact on people's quality life – this means affecting your ability to participate in tasks and activities that are important to them such as looking after yourself or completing daily activities.

Thinking about what is important to you, what do you think exercise staff should ask about when it comes to self care and Daily tasks?

3b. Quality of life can also include a persons ability to participate in hobbies, work or activities that connect them with family, friends and the community - what is important to you in relation to these activities?

3c. is there anything else you feel contributed to your quality of life that we have not discussed?

3d. What changes were or would be most important to you to feel like exercise sessions were helping you?

Section S1.4: Telehealth

Background and focused question

So, we would now like to briefly talk about telehealth. Telehealth refers to an appointment with a healthcare provider by phone or video call. To give you an idea of what a telehealth appointments like – so here you can see what is looks like

4a. Have you had any experiences completing a test or physical assessment during a telehealth appointment in your home environment?

Prompt: if unsure how this would practically work. This may include sitting up and down in a chair or walking up and down a corridor in your home.

Prompt: (if extra time) can you describe what it was like to do those tests via telehealth?

4b. Would you feel comfortable completing exercise tests during a telehealth appointment like in the pictures?

4.c How confident are you with completing questionnaires from a link in an e-mail on a secure website?

Section S1.5: Closing question

To close our session together:

5. Is there anything we have not discussed today that you feel is an important measure of change during or following an exercise program?

Thank participants and close session.

Figure S1: ICF framework domains, chapters, and outcomes.

Chapter name	First Level		Second Level	
	ICF outcome	ICF code	ICF Outcome	ICF code
Learning and applying knowledge	Learning and applying knowledge, unspecified	D199		
General tasks and demands	Undertaking a multiple tasks	D210		
	Carrying out daily routine	D230		
Mobility	Changing basic body position	D410	Sitting	D4103
	Changing basic body position	D410	Standing	D4104
	Transferring oneself	D420	Transferring oneself one sitting	D4200
	Lifting and carrying objects	D430	Lifting	D4300
			Carrying in the hands	D4301
			Carrying in the arms	D4302
	Walking	D450	Walking short distances	D4500
			Walking long distances	D4501
	Moving around	D455	Climbing	D4551
			Running	D4552
	Moving around in different locations	D460	Moving around within the home	D4600
			Moving around within buildings other than home	D4601
	Using transportation	D470		
	Driving	D475		
Self-Care	Washing oneself	D510		
	Looking after ones own health	D570		
Domestic Life	Acquisition of goods and services	D620	Shopping	D6200
	Preparing meals	D630		
	Doing housework	D640	garments	D6400
	Caring for household objects	D650	outdoors	D6505
	Assisting others	D660		
Interpersonal interactions and relationships	Complex interpersonal connections	D710		
	Informal social relationships	D750	Informal relationships with friends	D7500
	Family relationships	D760	Parent-child relationships	D7600
Major life areas	Reunerative employement	D850		
Community, social and civic life	Recreation and leisure	D920	Socializing	D9205

(a) Activities and Participation

Chapter name	First Level		Second Level	
	ICF outcome	ICF code	ICF Outcome	ICF code
Mental functions	Energy and drive	B130	Motivation	B1301
			Appetite	B1302
	Sleep functions	B135		
	Emotional functions	B152	Regulation of emotions	B1521
	Higher-level cognitive functions	B164		
Sensory functions and pain	Taste function	B250		
	Touch function	B265		
	Sensation of pain	B280		
Functions of the cardiovascular, haematological, immunological and respiratory systems	Blood pressure functions	B410	Maintenance of blood pressure	B402
	Heart functions	B410	Heart rate	B4100
	Haematological system functions	B430	Oxygen-carrying functions of the blood	B4301
	Respiratory functions	B440		
	Respiratory muscle functions	B445		
	Additional functions of the respiratory system	B450	Functions of coughing	B4501
	Exercise tolerance functions	B455	General physical endurance	B4550
			Aerobic capacity	B4551
			Fatiguability	B4552
	Sensations associated with cardiovascular and respiratory functions	B460		
Functions of the digestive, metabolic and endocrine systems	Weight maintenance functions	B530		
	Sensations associated with the digestive system	B535	Sensation of nausea	B5350
Neuromusculoskeletal and movement-related functions	Muscle endurance functions	B730	Power of muscles of all limbs	B7304
	Muscle endurance functions	B740	Endurance of muscle groups	B7401

(b)Body Functions

Chapter name	First Level		Second Level	
	ICF outcome	ICF code	ICF Outcome	ICF code
Structure of nervous system	Structure of brain	S110		
Structures related to movement	Additional musculoskeletal structures related to movement	S770	Bones	S7701
Structures related to movement	Additional musculoskeletal structures related to movement	S770	Muscles	S7702

(c) Body Structures

Chapter name	First Level		Second Level	
	ICF outcome	ICF code	ICF Outcome	ICF code
Products and Technology	Products or substance for personal consumption	E110	E1101 Drugs	E1101
	Products and technology for personal indoor and outdoor mobility and transportation	E120	General products and technology for personal indoor and outdoor mobility and transportation	e1200
Support and relationships	Immediate family	E310		
	Friends	E320		
Services, systems and policies	Health services, systems and policies	E580	Health systems	e5801

(d) Environmental Factors
